# The result of prospective evaluation of 3-dimensional printing–aided extensive thoracoabdominal aorta repair

**DOI:** 10.1016/j.xjtc.2023.04.011

**Published:** 2023-05-08

**Authors:** Sung Jun Park, Jin Kyung Kim, Hong Rae Kim, Taehun Kim, Sangwook Lee, Guk Bae Kim, Dong Hyun Yang, Joon Bum Kim

**Affiliations:** a Department of Thoracic and Cardiovascular Surgery, Severance Cardiovascular Hospital, Yonsei University College of Medicine, Seoul, Korea; b Department of Thoracic and Cardiovascular Surgery, University of Ulsan College of Medicine, Asan Medical Center, Seoul, Korea; c Department of Convergence Medicine, Asan Medical Institute of Convergence Science and Technology, University of Ulsan, Asan Medical Center, Seoul, Korea; d Anymedi Inc (Product R&D Center), Seoul, Korea; e Department of Radiology, University of Ulsan College of Medicine, Asan Medical Center, Seoul, Korea

**Keywords:** thoracoabdominal aortic aneurysm, 3D printing, paraplegia, intercostal artery, segmental artery

## Abstract

**Objectives:**

Paraplegia is a distressing complication after open thoracoabdominal aortic aneurysm (TAAA) repair, and revascularization of T8-L2–level segmental arteries is considered pivotal to prevent paraplegia. We employed 3-dimensional (3D) printing to efficiently revascularize segmental/visceral arteries and prospectively evaluated its safety and efficacy.

**Methods:**

From January 1, 2020, to June 30, 2022, we prospectively enrolled patients of extent I, II, or III TAAA repair. Guidance models were 3D-printed based on preoperative computed tomography, and multibranched aortic grafts were manually constructed upon this model before surgery. The composite outcome of operative mortality, permanent stroke, and permanent spinal cord deficit (SCD) was compared with the historical control group (n = 77, in 2015-2020), subjected to similar TAAA repair without 3D printing.

**Results:**

A total of 38 patients (58.6 ± 13.2 years) underwent open TAAA repair with the aid of 3D printing. Extent I, II, and III repairs were performed in 14 (36.8%), 17 (44.7%), and 7 (18.4%), respectively. Concomitant arch repair and bi-iliac reconstruction were performed in 7 (18.4%) and 6 patients (15.8%), respectively. Mean pump time was 107.7 ± 55.5 minutes. Operative mortality, permanent stroke, and permanent SCD each occurred in 1 patient (2.6%), and the incidence of the composite outcome was 7.9% (3/38). In the control group, mean pump time was 166.0 ± 83.9 minutes, significantly longer than the 3D-printing group (*P* < .001), and operative mortality, permanent stroke, permanent SCD, and the composite outcome occurred in 7 (9.1%), 9 (11.7%), 8 (10.4%), and 19 (24.7%), respectively.

**Conclusions:**

Open repairs of extensive TAAA with 3D printing showed favorable safety and efficacy, which need further validation by larger studies.

## Graphical abstract

**Figure:**
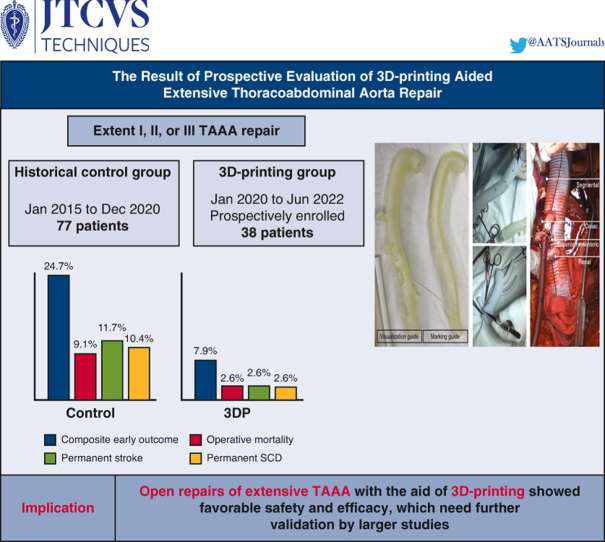

**Figure:**
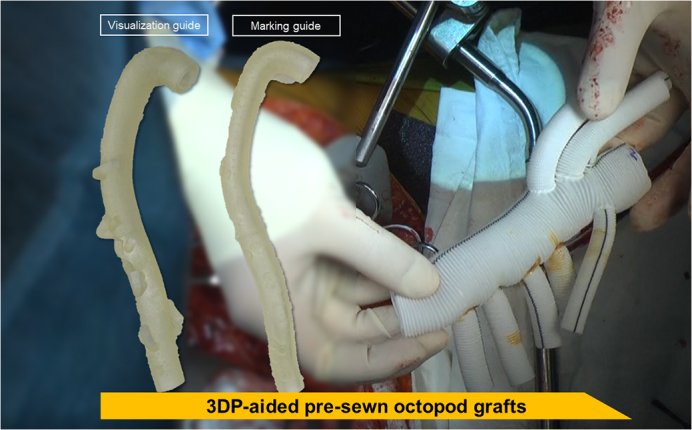
3DP-aided octopod grafts were prepared before surgery using the 3D-printed guides.

**Central Message**

This prospective evaluation of 3DP-aided TAAA repair demonstrated favorable safety and efficacy profiles. These results need to be validated by larger multicenter studies to gain generalizability.

**Perspective**

Postoperative paraplegia is a distressing complication after open TAAA repair, and revascularization of T8-L2–level segmental arteries is regarded pivotal to prevent paraplegia. The 3DP-aided octopod technique can promote efficient segmental artery revascularization, which may contribute to improved results.

Postoperative paraplegia is a distressing complication of open thoracoabdominal aortic aneurysm (TAAA) repair, and the development of this complication is rather unpredictable, even with optimal intra-/perioperative management. Although revascularization of T8-L2–level segmental arteries is considered pivotal in preventing paraplegia, this procedure is frequently complicated by high anatomical variability among patients.^1^, ^2^, ^3^ It is also not uncommon for the segmental arteries to be severely dislocated or technically challenging to designate a level, particularly in the case of a tortuous or severely dilated aorta. In addition, the quality of surrounding tissues is frequently compromised by severe atherosclerosis or calcification.

Despite outstanding advancements in this type of surgery over the past decades, significant improvements in the incidence of postoperative paraplegia could not be seen.^4^, ^5^, ^6^ In a focused effort to reduce postoperative paraplegia after open TAAA repair, we aimed to completely revascularize all the T8-L12–level segmental arteries, which were identified and targeted before surgery based on preoperative computed tomography (CT) images. However, owing to the considerable surgical burden of extensive segmental artery revascularization, excessive time consumption and associated risks can be imposed. To offset this surgical burden, we have been employing the octopod technique for extensive TAAA repair since 2015. This technique has evolved with the aid of 3-dimensional printing (3DP) technology, and the initial results were reported previously.^7^^,^^8^

In this study, we sought to evaluate the outcomes of the 3DP-aided octopod technique by using a prospective design. Furthermore, to validate the safety and efficacy, an additional comparison was made with a historical control group subjected to similar TAAA repair but without the 3DP technique.

## Methods

### Patient Enrollment and Outcomes of Interest

This is a prospective, open-label, single-arm trial that evaluated the operative outcomes of open TAAA repair. We enrolled patients who required extent I, II, or III open TAAA repair between January 1, 2020, and June 30, 2022. Patients who did not require T8-L12 segmental artery anastomosis based on the preoperative CT scans were excluded. This study was reviewed and approved by the Asan Medical Center Institutional Review Board (study number: 2020-0030; approval date: January 6, 2020). Informed written consent for publication of study data was obtained from each patient who underwent open TAAA repair with 3DP-aided octopod technique. Requirements for informed consent were waived for the historical control group due to the retrospective nature of the study, as directed by the institutional review board.

Data on historical control group patients were drawn from the institutional cardiac surgery database. These patients underwent elective extent I, II, or III open TAAA repair without octopod technique in Asan Medical Center from January 2015 to December 2020. This control group was created to serve as reference results for open TAAA repair for validation of safety profiles.

The primary outcome was the composite early outcome, defined as the composite of operative mortality (30-day mortality or in-hospital mortality), permanent disabling stroke, and permanent spinal cord deficit (SCD). The secondary outcomes were the individual components of the primary composite outcome: operative mortality, permanent disabling stroke, and permanent SCD. The primary and secondary outcomes and early postoperative complications were also defined as events that occurred within 30 days of surgery or during index hospitalization. Permanent disabling stroke was defined as stroke with any functional or neurologic sequelae, confirmed by an experienced neurologists and using brain imaging modalities, such as CT scan or magnetic resonance imaging. Permanent SCD excluded temporary paraplegia or paraparesis resolved during index hospitalization without any functional or neurologic sequelae.

### Preparation of the Octopod Graft With the Aid of 3DP Technology

The detailed procedure for octopod graft preparation has been described previously.^7^^,^^8^ For revascularization of targeted segmental arteries, 10- to 16-mm grafts (HEMASHIELD Platinum straight graft; MAQUET Cardiovascular LLC) were individually anastomosed to a commercially available 4-branched TAAA graft (Coselli thoracoabdominal graft; Vascutek Ltd) in the operating theater during anesthesia induction of patients. The side branches of a TAAA graft were used for visceral revascularization (celiac trunk, superior mesenteric artery, and right/left renal arteries) in most cases, owing to the homogenous anatomy in the origins of visceral arteries. The 3DP-aided octopod graft was prepared through these steps:

1. The framework of the aortic graft was modeled based on the center line of a 3D-reconstructed native aorta rendered by an electrocardiography-gated CT scan (Figure 1).

**Figure 1:**
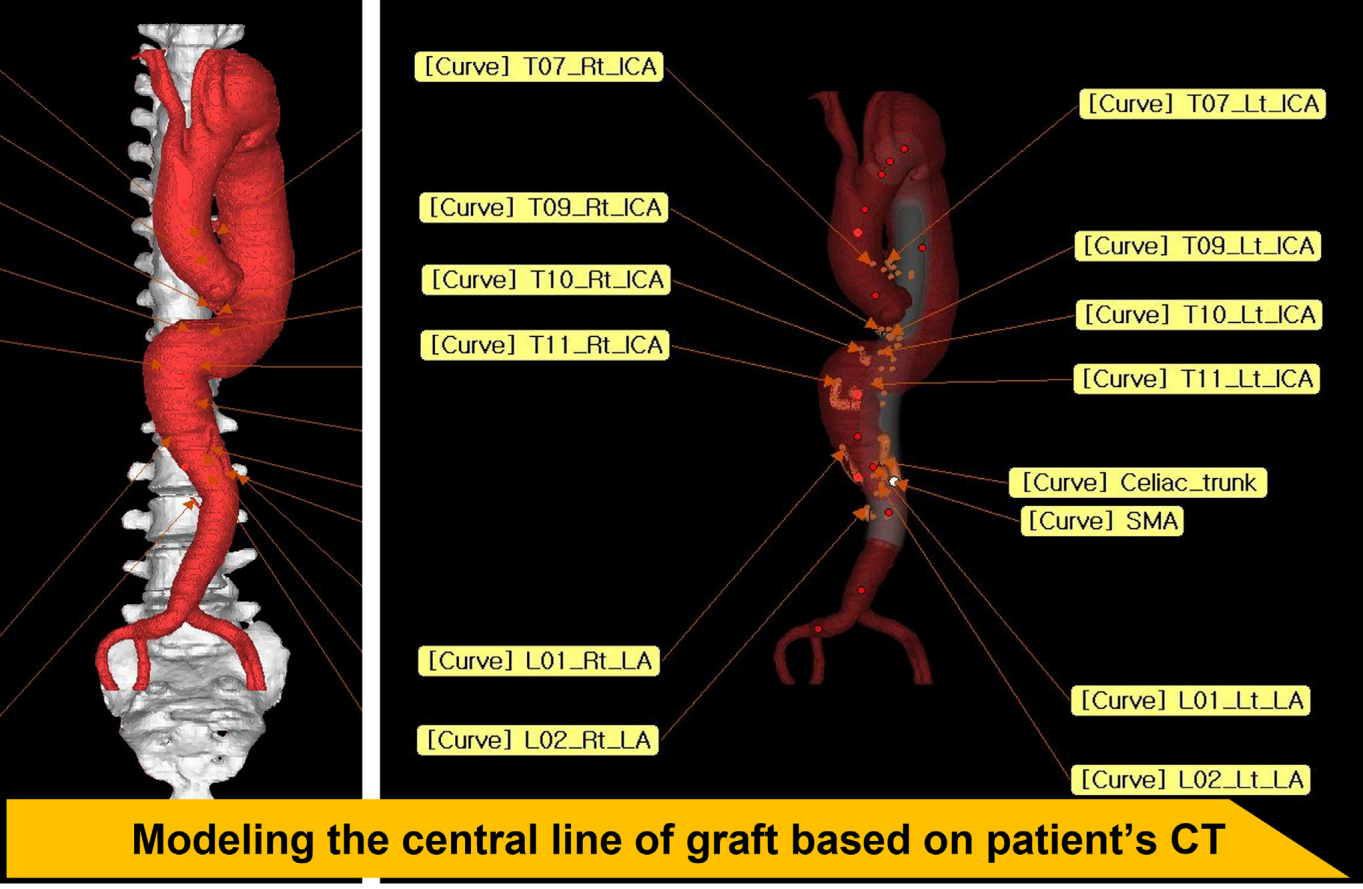
Three-dimensional modeling process for the aortic graft framework. *Rt*, Right; *ICA*, intecostal artery; *Lt*, left; *SMA*, superior mesenteric artery; *LA*, lumbar artery; *CT*, computed tomography.
2. The final version of the 3D aortic graft model incorporating segmental and visceral arteries was mutually created by the operator and 3DP manufacturer (Anymedi Inc) (Figure 1).
3. The aortic graft model was printed out as 2 guides (visualization and marking) (Figure 2, *A*). It takes approximately 3 days to 2 weeks from acquiring the patient's CT image to receiving the 3D-printed models, and the required period can be adjusted according to the patient's emergency. The marking guide was inserted into the TAAA graft while manually constructing the octopod graft, so that the spatial relationship between the branch vessels can be easily and comfortably identified using a small protuberance (Figure 2, *B*). The details of the printing technology are described in our previous publications.^8^^,^^9^

**Figure 2:**
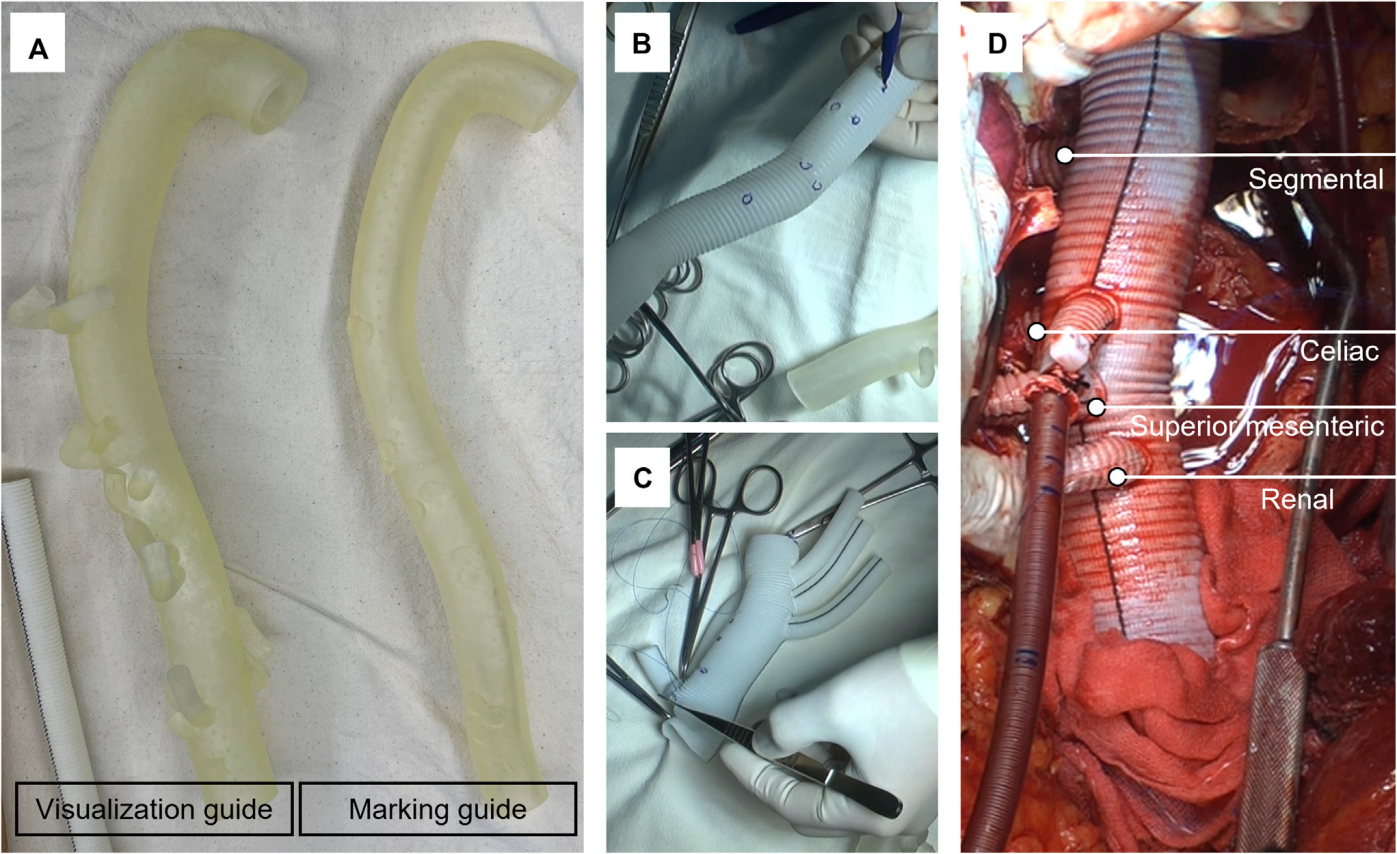
Application of 3DP guidance octopod graft for thoracoabdominal aortic aneurysm repair. A, Two forms of aortic graft model: visualization guide and marking guide. B, The marking guide is inserted into the graft, and branch vessels can be easily identified. C, Using a marking guide, the 3DP-aided octopod grafts were manually constructed. D, Intraoperative findings.
4. Using the marking guide, the 3DP-aided octopod grafts were manually constructed (Figure 2, *C*). When the marking guide was aligned into the TAAA graft, the position of the segmental arteries was determined by fully overstretching the TAAA graft using the celiac trunk as a reference point (Figure 2, *D*).

### Operative Techniques

Our operative technique for open TAAA repair has been described in our previous study.^8^ Previously, we routinely inserted a cerebrospinal fluid drainage catheter in all patients undergoing extensive TAAA repair (ie, Crawford extent II and III). However, cerebrospinal fluid drainage was not performed in this study because of serious procedure-related complications, such as trauma-related myelopathy and spinal cord infection. In brief, after full exposure of the operative field and meticulous bleeding control, cardiopulmonary bypass was established using the left femoral artery and vein. Permissive hypothermia (nasopharyngeal temperature, 31 °C) was the main principle to prevent cardiac arrest, but moderate hypothermia (nasopharyngeal temperature, 25-28 °C) was used when proximal aortic clamping was not feasible. Visceral and renal protective procedures were performed as described in our previous studies.^8^ The celiac axis and superior mesenteric artery were selectively perfused with normothermic blood. For renal protection, multiple boluses of cold lactated Ringer's solution (4 °C) were directly delivered to the left and right renal arteries every 15 to 20 minutes; however, this procedure has recently been simplified to a single administration of histidine-tryptophan-ketoglutarate solution.

For individual anastomosis of segmental arteries, a pair of segmental artery orifices were trimmed into an oval-shaped button with a margin of 10 mm. In earlier, this button was anastomosed to a 10-mm branch of octopod graft, but later 16-mm grafts were used for segmental reconstruction for a more natural graft configuration (Figure 2, *D*). Small intercostal artery orifices that were not seen on CT were not incorporated into repair and were over-sewn with pledgeted mattress sutures using 3-0 polypropylene.

### Statistical Analysis

Categorical variables were presented as frequencies and percentages and compared using the χ^2^ test or Fisher exact test. Continuous variables with normal distribution were expressed as mean ± standard deviation and compared using the Student *t*-test. Continuous variables with nonnormal distribution were expressed as median [interquartile rage] and compared using the Wilcoxon-rank sum test. Among continuous variables, creatinine level and lower body ischemic time showed nonnormal distribution, examined by the Kolmogorov–Smirnov test and Shapiro–Wilk test. All reported *P* values are 2-sided. R software, version 4.1.0 (R Foundation) was used for the statistical analysis.

## Results

### Patient Characteristics

A total of 38 patients underwent open TAAA repair using the 3DP-aided octopod technique. Baseline patient characteristics are described in Table 1. The mean patient age was 58.6 ± 13.2 years. Genetic aortopathy was present in 7 patients (18.4%), 6 (15.8%) of whom had Marfan syndrome. Of the patients, 52.6% (20/38) had undergone previous aortic surgery and 2 (5.3%) had a history of previous thoracic endovascular aortic repair. Chronic aortic dissection was the most common aortic pathology in 21 patients (55.3%), and chronic degenerative aneurysm was observed in 13 patients (34.2%). The preoperative maximal aortic diameter was 58.97 ± 15.30 mm.

**Table 1:** Baseline characteristics of patients with 3DP-aided octopod technique and the historical control group

Variables	3DP-aided Octopod technique	Control group	*P* value
	N = 38	N = 77	
Age, y	58.6 ± 13.2	59.0 ± 13.1	.90
Female	10 (26.3)	26 (33.8)	.55
Body mass index, kg/m^2^	24.97 ± 4.84	24.56 ± 4.39	.65
Marfan syndrome	6 (15.8)	18 (23.4)	.49
Other genetic aortopathy	1 (2.6)	1 (1.3)	.99
Hypertension	27 (71.1)	67 (87.0)	.07
Diabetes mellitus	3 (7.9)	10 (13.0)	.62
Current smoking	7 (18.4)	12 (15.6)	.91
Past smoking	13 (34.2)	25 (32.5)	.99
Chronic lung disease	1 (2.6)	6 (7.8)	.50
End-stage renal disease	2 (5.3)	5 (6.5)	.99
Previous aorta surgery	20 (52.6)	48 (62.3)	.43
Previous thoracic endovascular aortic repair	2 (5.3)	2 (2.6)	.85
Previous percutaneous coronary intervention	3 (7.9)	4 (5.2)	.88
Previous coronary artery bypass grafting	4 (10.5)	9 (11.7)	.99
Stroke	2 (5.3)	5 (6.5)	.99
Hemoglobin, g/dL	12.76 ± 1.79	12.71 ± 1.72	.89
Creatinine, mg/dL	0.98 [0.90, 1.14]	0.90 [0.76, 1.22]	.21
Symptomatic	18 (47.4)	18 (23.4)	.017
Concealed rupture	1 (2.6)	0 (0)	.72
Subacute aortic dissection	3 (7.9)	0 (0)	.06
Chronic aortic dissection	21 (55.3)	53 (68.8)	.22
Chronic degenerative aneurysm	13 (34.2)	24 (31.2)	.91
Maximal diameter, mm	58.97 ± 15.30	58.92 ± 11.26	.47

Data are mean ± SD, median [interquartile rage], or numbers (percentages). *3DP*, 3-Dimensional printing.

A total of 77 patients were included in the historical control group. Consecutive elective cases without the octopod technique were selected for this control group. Baseline profiles were generally similar between the 3DP and control groups, except that there were less-symptomatic patients (23.4%) in the control group, whereas there were 3 (47.4%%) in the 3DP group (*P* = .002).

### Operative Profiles of 3DP-Aided TAAA Repair

Operative profiles are summarized in Table 2 (left column). Extent I, II, and III repairs were performed in 14 (36.8%), 17 (44.7%), and 7 (18.4%) patients, respectively. Concomitant aortic arch repair or iliac artery reconstruction was required in 8 (21.1%) and 6 (15.8%) patients, respectively. Reattachment of segmental arteries was performed in 36 patients (94.7%). Total circulatory arrest was required in 12 patients (31.6%) for proximal anastomosis when proximal clamping was not feasible.

**Table 2:** Operative profiles of patients with 3DP-aided octopod technique and the historical control group

Variables	3DP-aided Octopod technique	Control group	*P* value
	N = 38	N = 77	
Cerebrospinal fluid drainage	0 (0)	49 (63.6)	<.001
Previous aortic surgery through thoracotomy	6 (15.8)	3 (3.9)	.06
Crawford extent			.021
Extent I	14 (36.8)	48 (62.3)	.017
Extent II	17 (44.7)	17 (22.1)	.022
Extent III	7 (18.4)	12 (15.6)	.91
Concomitant arch repair	8 (21.1)	11 (14.3)	.514
Concomitant iliac artery reconstruction	6 (15.8)	2 (2.6)	.046
Completion elephant trunk	2 (5.3)	4 (5.2)	.99
Segmental artery reattachment	36 (94.7)	46 (59.7)	<.001
Number of anastomoses	2.00 [2.00, 3.00]	Unknown	N/A
Celiac trunk anastomosis	24 (63.2)	38 (49.4)	.23
Superior mesenteric artery anastomosis	24 (63.2)	27 (35.1)	.008
Right renal anastomosis	23 (60.5)	26 (33.8)	.011
Left renal anastomosis	23 (60.5)	25 (32.5)	.008
Splenectomy	0 (0)	2 (2.6)	.81
Body temperature	28.76 ± 7.53	27.96 ± 3.92	.002
Cardiopulmonary bypass time, min	107.7 ± 55.5	166.0 ± 83.9	<.001
Requiring total circulatory arrest	12 (31.6)	29 (37.7)	.67
Total circulatory arrest time, min	11.8 ± 6.1	15.1 ± 7.3	.19
Requiring lower body ischemia	31 (81.6)	57 (74.0)	.51
Lower body ischemic time, min	9.3 [7.0, 13.5]	13.0 [10.0, 19.0]	.001

Data are mean ± SD, median [interquartile rage], or numbers (percentages). *3DP*, 3-Dimensional printing; *N/A*, not available.

### Operative Profiles of Control Group

Operative profiles are summarized in Table 2 (right column). Extent I, II, and III repairs were performed in 48 (62.3%), 17 (22.1%), and 12 (15.6%) patients, respectively. The proportion of extent I repair was significantly greater in the control group (*P* = .017) and significantly lower in the case of extent II (*P* = .022). Branch vessel anastomosis, including segmental artery (*P* < .001), superior mesenteric artery (*P* = .008), right renal artery (*P* = .011), and left renal artery (*P* = .008), was more frequent in the 3DP group. As compared with the control group, the 3DP group had significantly shorter time profiles, which included mean cardiopulmonary bypass time (107.7 ± 55.5 minutes vs 166.0 ± 83.9 minutes, *P* < .001) and median lower body ischemia time (9.3 minutes vs 13.0 minutes, *P* = .001).

### Operative Outcomes

In the 3DP group, operative mortality occurred in 1 patient (2.6%) (Table 3, left column). In this case, a retrograde type A dissection occurred during extent I TAAA repair; thus, an exigent ascending aorta and hemiarch replacement was promptly performed. However, the patient died from intractable bleeding on the first postoperative day. There were 6 cerebral complications (15.8%) after surgery, which included 3 cases of ischemic stroke, 1 case of subdural hemorrhage, and 2 cases of seizure. Among these cerebral events, only 1 patient (2.6%), who underwent concomitant total-arch replacement, required rehabilitative therapy and transfer to a nursing hospital because of an embolic infarct in the middle cerebral artery. The other 5 patients recovered and were discharged without sequelae. Permanent paraplegia also occurred in 1 patient (2.6%), but there were no temporary SCD cases.

**Table 3:** Early operative outcomes of patients with 3DP-aided octopod technique and the historical control group

Variables	3DP-aided octopod technique	Control group	*P* value
	N = 38	N = 77	
Composite early outcome	3 (7.9)	19 (24.7)	.06
Operative mortality	1 (2.6)	7 (9.1)	.37
Cerebral complications (composite)	6 (15.8)	9 (11.7)	.75
Ischemic stroke	3 (7.9)	8 (10.4)	.93
Subdural hemorrhage	1 (2.6)	2 (2.6)	.99
Seizure	2 (5.3)	4 (5.2)	.9
Permanent disabling stroke	1 (2.6)	9 (11.7)	.20
Spinal cord deficit (composite)	1 (2.6)	11 (14.3)	.11
Permanent spinal cord deficit	1 (2.6)	8 (10.4)	.28
Paraplegia	1 (2.6)	6 (7.8)	.50
Paraparesis	0 (0.0)	2 (2.6)	.81
Temporary spinal cord deficit	0 (0.0)	3 (3.9)	.54
Paraplegia	0 (0.0)	2 (2.6)	.81
Paraparesis	0 (0.0)	1 (1.3)	.99
Bowel ischemia	0 (0.0)	1 (1.3)	.99
Acute renal dysfunction	3 (7.9)	15 (19.5)	.18
New-onset dialysis	1 (2.6)	13 (16.9)	.06
Exploration for bleeding	3 (7.9)	1 (1.3)	.20
Postoperative mechanical support (ECMO)	0 (0)	4 (5.2)	.37
Wound problem	0 (0)	4 (5.2)	.37
Pneumonia	5 (13.2)	12 (15.6)	.95
Pericardiocentesis	0 (0)	4 (5.2)	.37
Tracheostomy	3 (7.9)	11 (14.3)	.50
Hoarseness	6 (15.8)	13 (16.9)	.99
Mechanical ventilation >48 h	17 (44.7)	24 (31.2)	.22
Intensive care unit stay	4.50 [2.25, 7.00]	4.00 [2.00, 7.00]	.30

Data are median [interquartile rage], or numbers (percentages). *3DP*, 3-Dimensional printing; *ECMO*, extracorporeal membrane oxygenation.

The operative outcomes of the control group are also described in Table 3 (right column). Operative death, permanent disabling stroke, and permanent SCD occurred in 7 (9.1%), 9 (11.7%), and 8 (10.4%) patients, respectively. The composite early outcome occurred in 19 patients (24.7%). There were no differences in operative outcomes between groups, including composite early outcome (*P* = .06), operative mortality (*P* = .37), permanent disabling stroke (*P* = .20), and permanent SCD (*P* = .28).

## Discussion

In this prospective trial, we evaluated the operative outcomes of the 3DP-aided octopod technique in open TAAA repair and confirmed its acceptable safety and efficacy. The operative mortality rate was 2.6%, and the incidence of disabling stroke and permanent SCD was also 2.6%. The 3DP group showed superior safety profiles compared with the historical control group, which did not use the technique. The 3DP-aided octopod technique was associated with a significantly lower risk of the composite outcome of operative mortality, disabling stroke, or permanent SCD. Individual components were also appeared more favorable; however, the differences were not statistically significant, owing to a small sample size in this study. In terms of efficacy, this technique was associated with shorter time profiles, despite more extensive surgery represented by frequent branch vessel revascularization.

Paraplegia may be the most distressing and overwhelming complication after extensive TAAA repair. Patients who survive with this complication often experience a reduced quality of life accompanied by extreme psychological burden. Considerable efforts have been made to reduce the occurrence of paraplegia, but its incidence remains at approximately 10% after extensive TAAA repair, even when surgery is performed in renowned aortic centers around world.^5^^,^^10^, ^11^, ^12^ Although permanent stroke is also an overwhelming complication, its incidence is relatively lower, and its symptoms or sequelae can be improved and compensated through rehabilitation therapy, unless the patient is in a comatose state. Therefore, the primary goal of TAAA repair is prevention of paraplegia occurrence.

Griepp and Griepp^13^ have revealed the importance of spinal cord circulation and the concept of the collateral network in their experimental studies. In particular, their experimental pig model revealed that the spinal cord perfusion pressure can be recovered reliably through the collateral networks a week after segmental artery sacrifice.^14^^,^^15^ It should be noted here that they did not claim to allow sacrificing segmental arteries in the belief of collateral networks. Rather, they proposed a 2-stage concept for extensive TAAA repair to reduce paraplegia, because segmental artery occlusion was decisive for the development of paraplegia in their experimental results.^16^^,^^17^ We believe that the concept of this study is grounded by their findings. Furthermore, we believe that this 3D model also can be used in the 2-stage concept proposed by Griepp and Griepp.

Ensuring sufficient spinal cord perfusion may be the core principle in paraplegia prevention. More specifically, the blood supply originates from T8-L2–level segmental arteries. The occurrence of paraplegia despite the reperfusion of segmental arteries at this level indicates that reperfusion was not sufficient. To avoid this complication, we focused primarily on providing sufficient blood supply through comprehensive and extensive revascularization of the T8-L2–level segmental arteries. However, extensive segmental revascularization is time-consuming because it involves multiple anastomoses, and it is difficult to determine the segmental arteries belonging to T8-L2 level in the surgical field. The 3DP technology enabled us to efficiently address these issues. Using the preoperative CT images, the T8-L2–level segmental arteries could be identified and targeted in advance to the surgery, and the branch vessels too small to be detectable in CT could be excluded. Performing graft-to-graft anastomosis at the most suitable location before surgery can significantly reduce the time spent during surgery, and an optimal graft configuration can prevent graft kinking.

In this era, in which endovascular aortic repair is already commonplace, the leaderships of interventional treatment, including developers and practitioners, are striving to secure better performance and thus expand the realm.^18^^,^^19^ Although the role and place of surgery are becoming narrower, the expectations for surgery are rising as much as those of interventional treatments with very low early morbidity and mortality. We should show our craftmanship and sophisticated performance that future patients and community may accept.

### Limitations

This study has several limitations. First, all surgeries were exclusively performed by a single surgeon, who is affiliated to a tertiary referral aortic center. Therefore, the outcomes may not be generalizable to other institutions. The operative outcomes of the control group should be understood only as a reference rather than a comparison group. Statistical adjustment may lead to misunderstandings or overinterpretations, because there are significant imbalances in important variables such as surgical period, surgeon factor, and operative profiles.

## Conclusions

**Figure 3:**
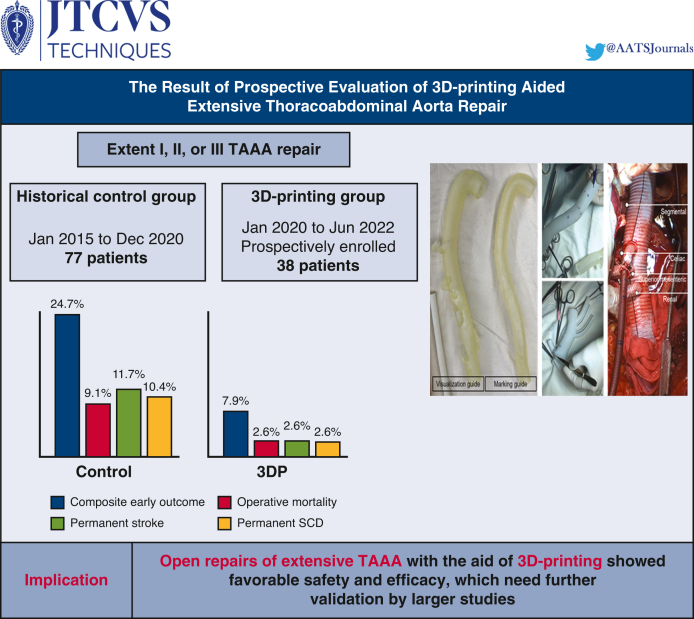
Open repairs of extensive thoracoabdominal aortic aneurysm with the aid of 3D-printing showed favorable safety and efficacy. *3D*, 3-Dimensional; *TAAA*, thoracoabdominal aortic aneurysm; *SCD*, spinal cord deficit; *3DP*, 3-dimensional printing.

TAAA repairs involving segmental artery revascularization were successfully conducted with the aid of 3DP guidance and showed favorable safety and efficacy profiles (Figure 3). However, multicenter prospective studies with a large cohort are required to validate the generalizability of our results.

### Webcast

You can watch a Webcast of this AATS meeting presentation by going to: https://www.aats.org/resources/the-result-of-prospective-evaluation-of-3-d-printing-guided-extensive-thoracoabdominal-aorta-repair.

**Figure:**
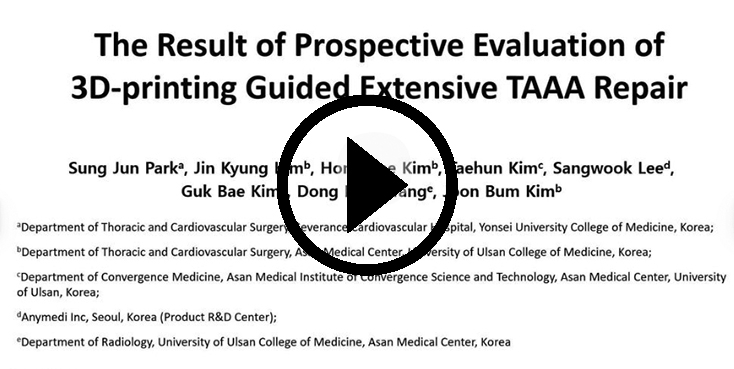

### Conflict of Interest Statement

Joon Bum Kim is a stockholder of Anymedi Inc. All other authors reported no conflicts of interest.

The *Journal* policy requires editors and reviewers to disclose conflicts of interest and to decline handling or reviewing manuscripts for which they may have a conflict of interest. The editors and reviewers of this article have no conflicts of interest.
